# No safe line: the human cost of dismantling LGBTQ+ youth support services

**DOI:** 10.1016/j.lana.2025.101191

**Published:** 2025-07-11

**Authors:** The Lancet Regional Health – Americas

**Editorial Disclaimer:***This editorial discusses suicide, mental health, and discrimination affecting LGBTQ+ youth*.

Imagine being a teenager, rejected by your family, bullied at school, and afraid to say who you are out loud. You gather the courage to call a helpline and someone who knows exactly what you are going through picks up. That connection might be the reason you survive the night. From July 17, 2025, that specialised, affirming, life-saving voice will no longer be available to LGBTQ+ youth in the USA as the press 3/PRIDE special helpline service—that provides support for LGBTQ+ youth over phone or text—will be discontinued. Following a series of dismantling and persecutory executive orders against LGBTQ+ people, the elimination of the press3/PRIDE special helpline is a public health crisis in the making.

Under the excuse that the 988 helpline will “focus on serving all help seekers”, the Trump administration has decided to discontinue press3/PRIDE. The federal Government's Substance Abuse and Mental Health Services Administration (SAMHSA) announced it will “will no longer silo LGB+ youth services,” suggesting they could get support from the general helpline and purposely ignoring the “T” for transgender people in the acronym. The decision, however, will eliminate a life-saving service created to protect a high-risk group of people. According to a 2024 Trevor Project survey, nearly half of trans/nonbinary youth (46%) seriously considered suicide within the past year. The CDC 2023 Youth Risk Behaviour Survey (YRBS) data showed 41% of LGBTQ+ students seriously considered suicide, compared to 13% of their cisgender and heterosexual colleagues. Since its implementation in 2022, trained counsellors with lived experience from the non-profit organisation that serves the press3/PRIDE special helpline have helped 1.3 million callers. In 2024 alone, counsellors supported 231,000 callers through the 988 line.

In the same 2023 YRBS, 65% of LGBTQ+ students reported feeling sad or hopeless, compared to 31% of cisgender and heterosexual students. The well accepted Minority Stress Model suggests that the internalisation of LGBTQ+ victimisation experiences and anti-LGBTQ+ messages can compound and produce negative mental health outcomes and increase suicide risk. LGBTQ+ young people who reported experiencing four types of minority stress (identity-based physical harm, discrimination, housing instability, and change attempts by parents) were found to be 12 times more likely to attempt suicide compared to youth who experienced none of these stress types. LGBTQ+ youth are not more prone to mental health diagnoses because of their gender identity; these are products of the social exclusion they face. As such, the mental health of young LGBTQ+ people must be considered through an intersectional lens, because overlapping identities will shape unique individual experiences. Young people experiencing multiple intersecting marginalised identities such as race, ethnicity, or being in foster care, juvenile justice, or experiencing homelessness, face multi-level systemic barriers that contribute to even poorer mental health outcomes.

Seeking mental health care is not easy. Shame, embarrassment, lack of trust, fear of being bullied, punished or abandoned, and a myriad of negative feelings preclude LGBTQ+ youth from sharing their feelings and asking for help even when they are at their lowest. For those with intersecting identities, the fear of not being fully understood is an additional barrier. In this sense, services such as the 988 helpline and other chat and text-based services can be important tools to overcome such barriers, especially when combined with affirmative and specialised support from people with lived experiences. Understanding the complexity of how multiple systems of oppression can affect LGBTQ+ youth and impact their mental health requires specialised attention. While professional support through formal healthcare assistance (such as psychiatric and psychological clinics) is imperative and essential, the role of the community, school-based services, and societal changes should not be taken lightly.

Acceptance is a powerful protective factor for suicidality in LGBTQ+ youth. Data shows that feelings of high social support from their families reduced suicidality rates by half in young LGBTQ+ people, and having at least one supportive adult in the family reduces suicidality rates significantly. Living in a supportive community has also proven protective for the mental health of LGBTQ+ youth, as have many school-based services. Sadly, since 2018, US state and federal legislatures have moved in the opposite direction, passing an unprecedented number of bills threatening the physical and mental health of LGBTQ+ youth, including banning gender affirming care, sports bans, restroom restrictions, and criminalisation of identities. In a 2024 report, nine out of ten LGBTQ+ young people said that recent politics negatively impacted their wellbeing, with two in five considering moving to another state because of restrictive bills passed. Data from the Healthy Minds study found that proposed anti-LGBTQ+ legislation was linked to increased depressive symptoms among LGBTQ+ college students.

The past 8 years have been especially hard for LGBTQ+ youth growing up in the USA. Instead of imposing legislation based on beliefs, the US Government should be legislating to make the country a better place for all its citizens. Creating affirming environments, ensuring access to supportive adults and peers, facilitating participation in affirming activities and clubs at school, respecting pronouns, enabling legal gender changes, and providing access to gender-affirming spaces and medical care have all been significantly associated with improved mental health and reduced suicide risk among LGBTQ+ youth.

The discontinuation of a successful public health service that acts as the last resource for a high-risk targeted group is not only cruel, but also sends a clear message that the Government is turning its back on those who need support the most. Protecting and caring for the mental health of LGBTQ+ youth should be a Government's priority. Building a society that accepts, respects, and loves people for who they are will lay the ground for our young people to thrive.

